# Supplementary material for: Common anti-cancer therapies induce somatic mutations in stem cells of healthy tissue
Source: Nat Commun. 2022 Oct 7;13:5915. doi: 10.1038/s41467-022-33663-5 (PMC9546852; doi:10.1038/s41467-022-33663-5)
Supplement: Supplementary file 1 — Supplementary information [file 41467_2022_33663_MOESM1_ESM.pdf]

Supplementary figure 1

a

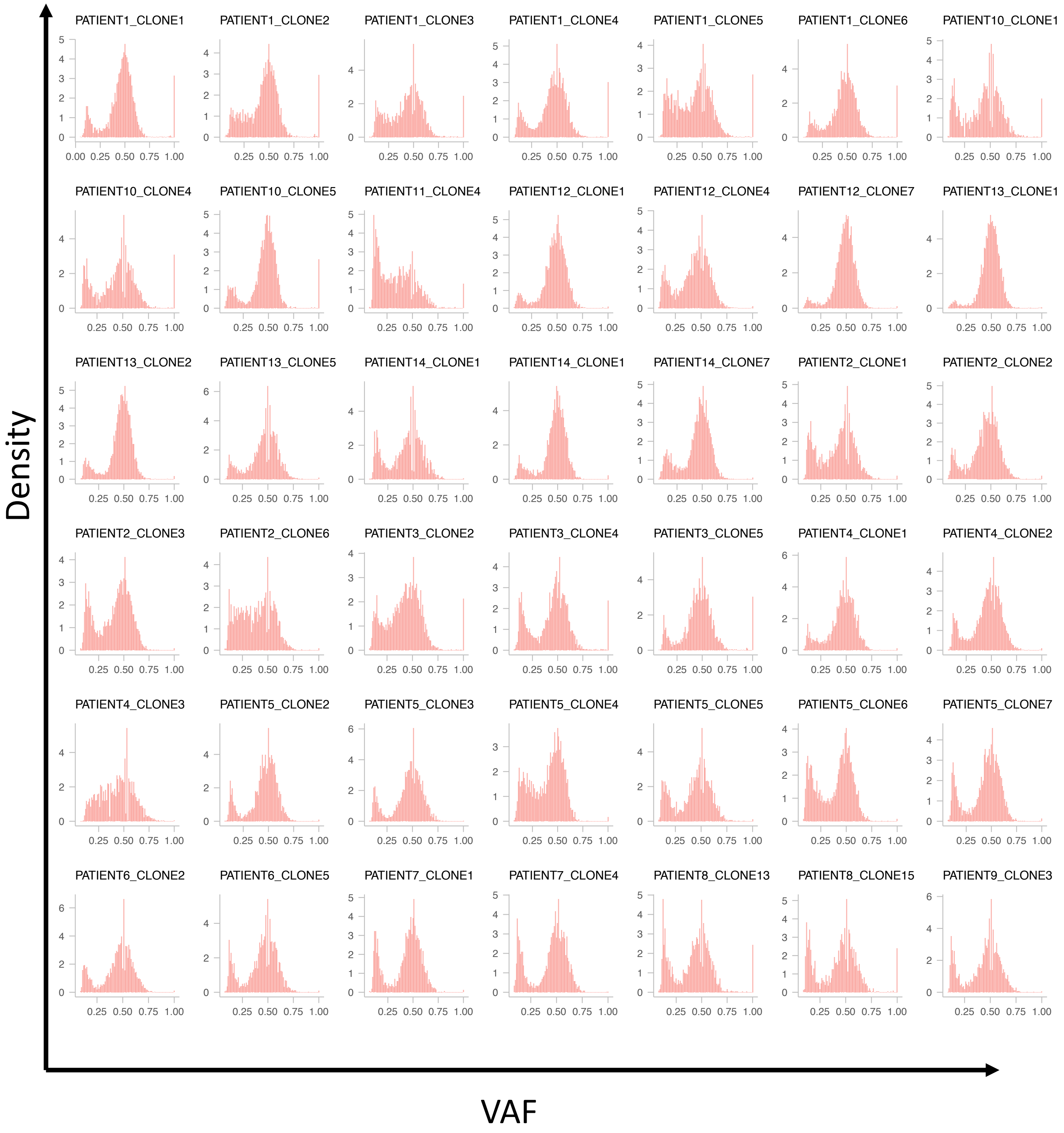

Supplementary Figure 1. Variant allele frequency distribution plot for each assessed ASC. A distribution plot of the VAFs of all somatic mutations that remain before VAF filtering. Clonal heterozygous somatic mutations form a peak around VAF = 0.5. A threshold of VAF ≥ 0.3 was used to obtain somatic mutations that were clonal in the organoid cultures and therefore present in the original cloned ASCs (see Methods). Mutations acquired after the single ASC expansion step are subclonal (that is, not present in all cells of the clonal culture) and have lower VAFs.

Supplementary figure 2

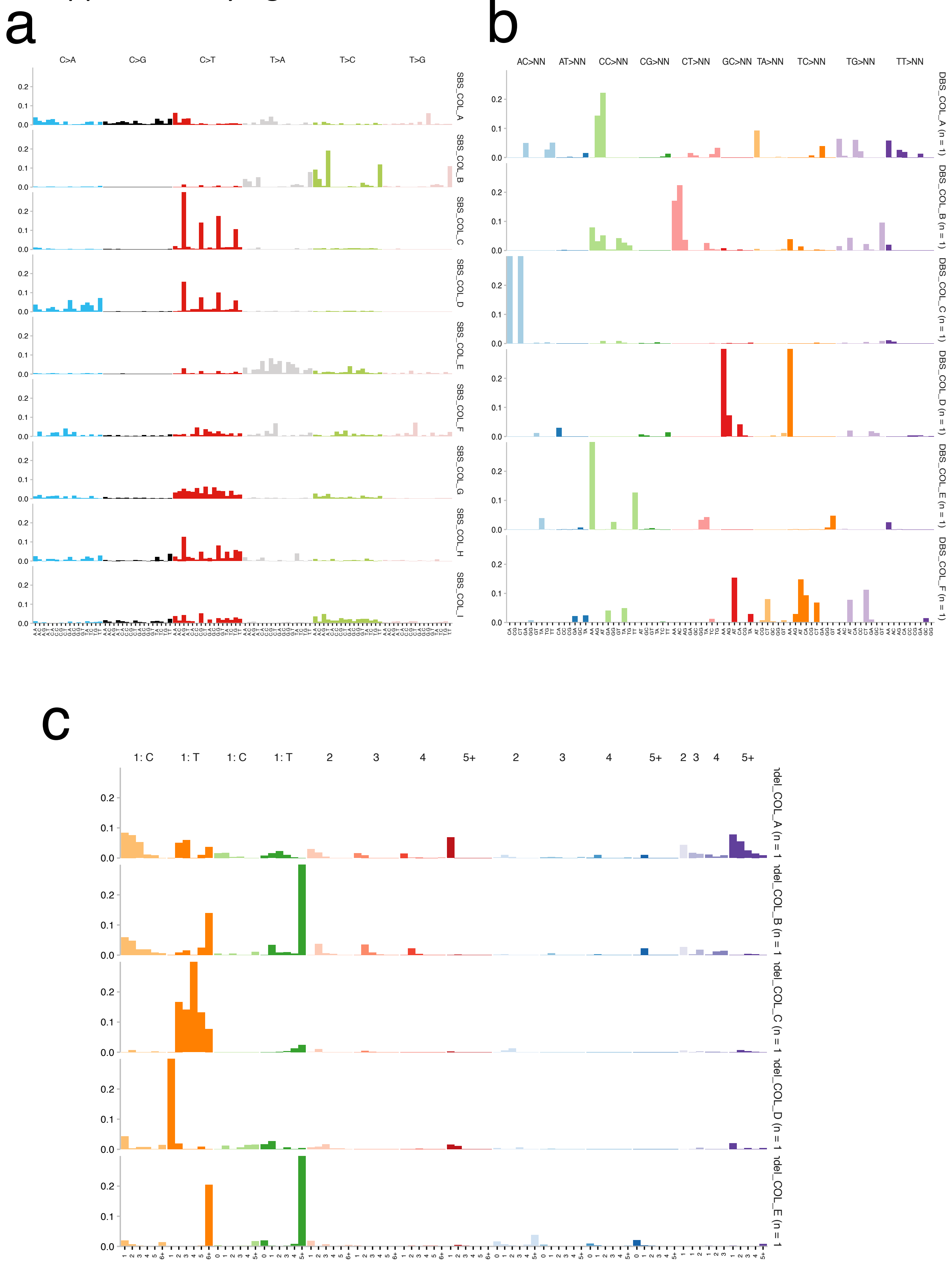

Supplementary Figure 2. ***De novo*** **signatures from treated and untreated colorectal tissue.** Mutational profiles (relative probability of each mutation context) of the (a) nine SBS, (b) six DBS and (c) five indel *de novo* mutational signatures from treated and untreated colorectal tissue. The signatures were extracted with Mutational Patterns on mutation data from 6 CapOx-treated colorectal donors (established in this study), 7 untreated donors[1], and a large mutation dataset of healthy colorectal tissue from Lee-Six[2]. The number of *de novo* signatures for the indicated mutation type was chosen by comparing the *de novo* extracted signatures from COSMIC mutational signatures and signatures from Lee-Six[2]., using a cosine similarity score of 0.8.

Supplementary figure 3

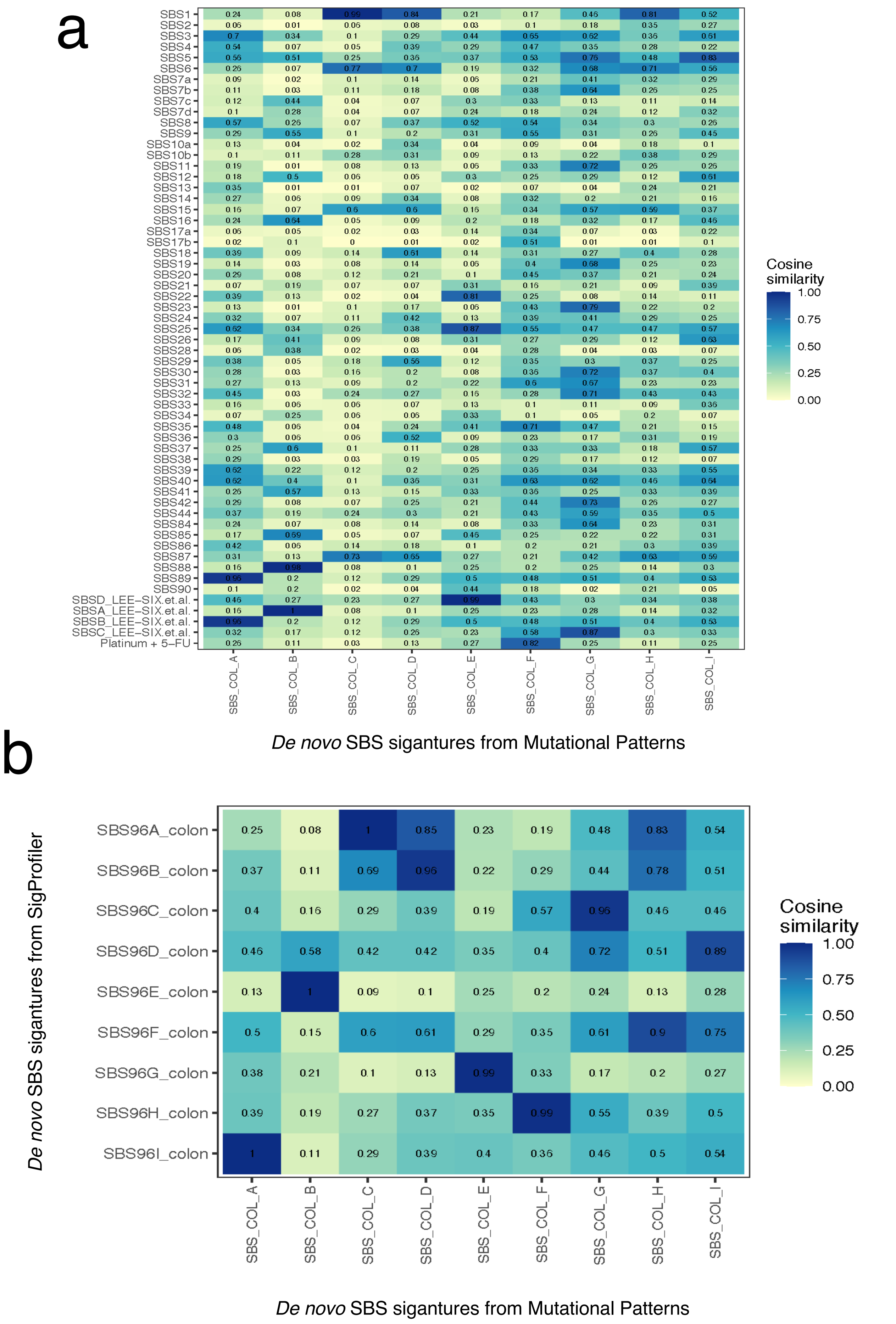

Supplementary Figure 3. ***De novo*** **SBS signature validation in colorectal tissue.** (a) Heatmap showing the cosine similarity scores for each *de novo* extracted colorectal SBS signature from Mutational Patterns with the COSMIC reference SBS signatures (as described in COSMIC: https://cancer.sanger.ac.uk/signatures/), the signatures from Lee-six[2], as well as the intermixed signature of 5-FU (SBS17) and platinum (SBS35) signature. Note that each of the nine *de novo* SBS signatures, except for SBS_COL_F, has a cosine similarity score of at least 0.8 with a COSMIC signature or with a signature from Lee-six[2]. SBS_COL_F, the distinct *de novo* signature, shows a cosine similarity score of 0.82 with the intermixed 5-FU (SBS17) and platinum (SBS35) signature. (b) Heatmap showing the cosine similarity scores for each *de novo* extracted colorectal SBS signature from Mutational Patterns with the *de novo* extracted colorectal SBS signature using SigProfiler. Note that SBS96H_colon from SigProfiler shows a cosine similarity of 0.99 with SBS_COL_F from Mutational Patterns.

Supplementary figure 4

a

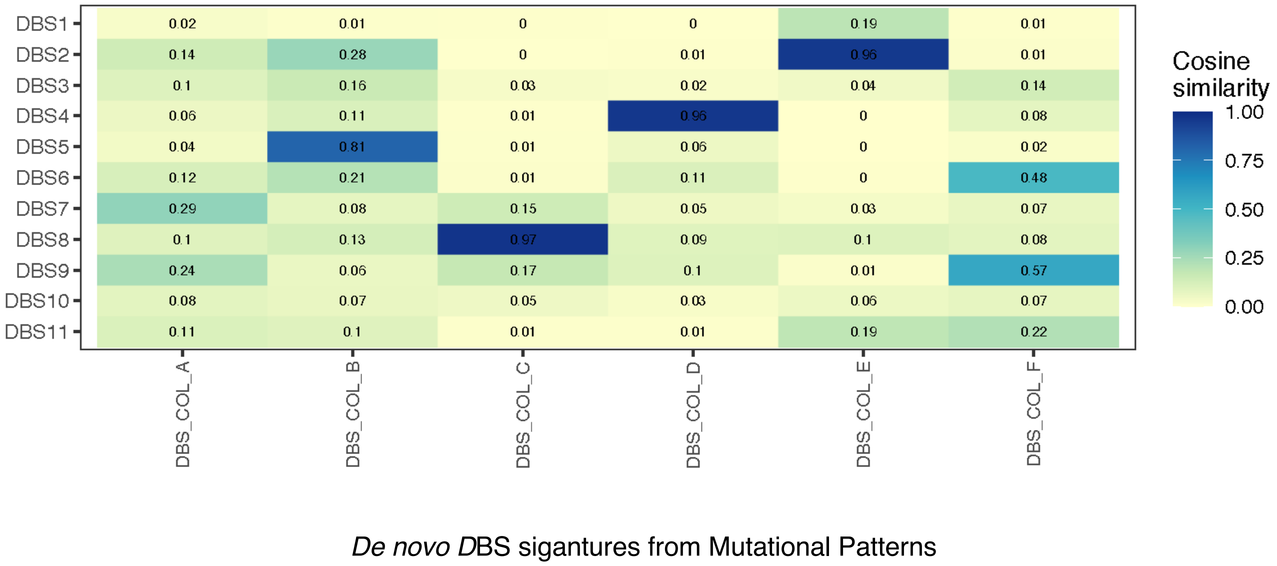

b

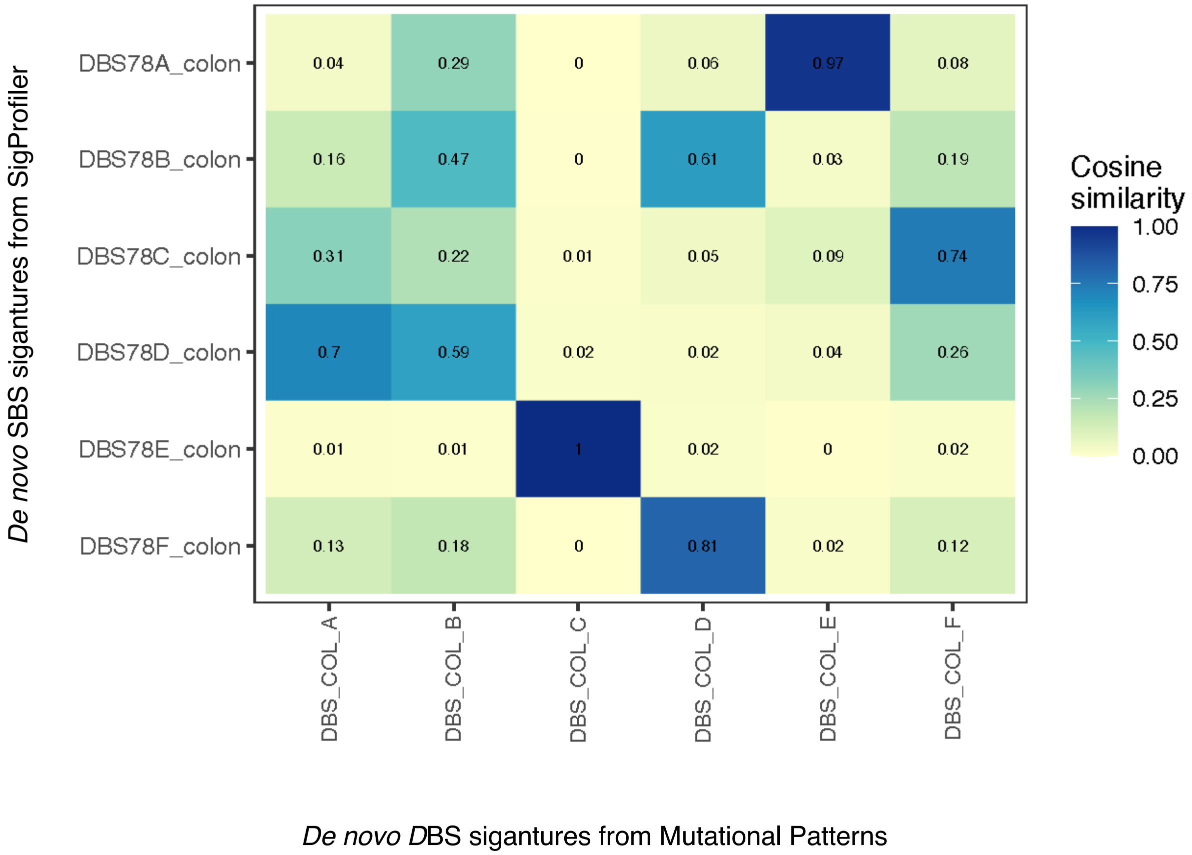

Supplementary Figure 4. *De novo* DBS signature validation in colorectal tissue. (a) Heatmap showing the cosine similarity scores for each *de novo* extracted colorectal DBS signature from Mutational Patterns with the COSMIC reference DBS signatures. (b) Heatmap showing the cosine similarity scores for each *de novo* extracted colorectal DBS signature from Mutational Patterns with the *de novo* extracted colorectal DBS signature from SigProfiler.

Supplementary figure 5

a

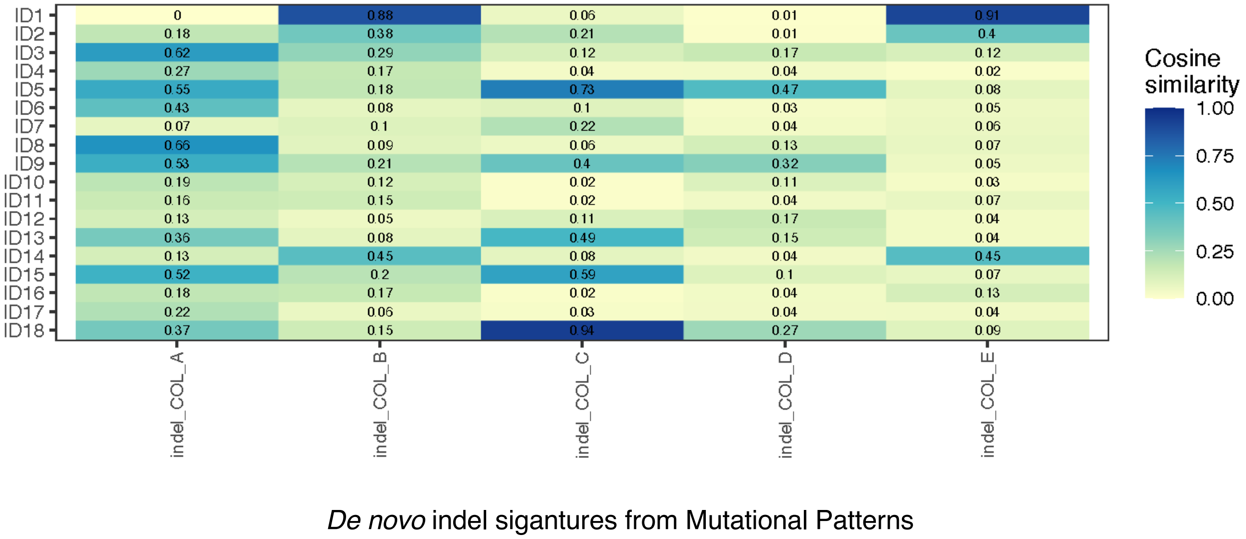

b

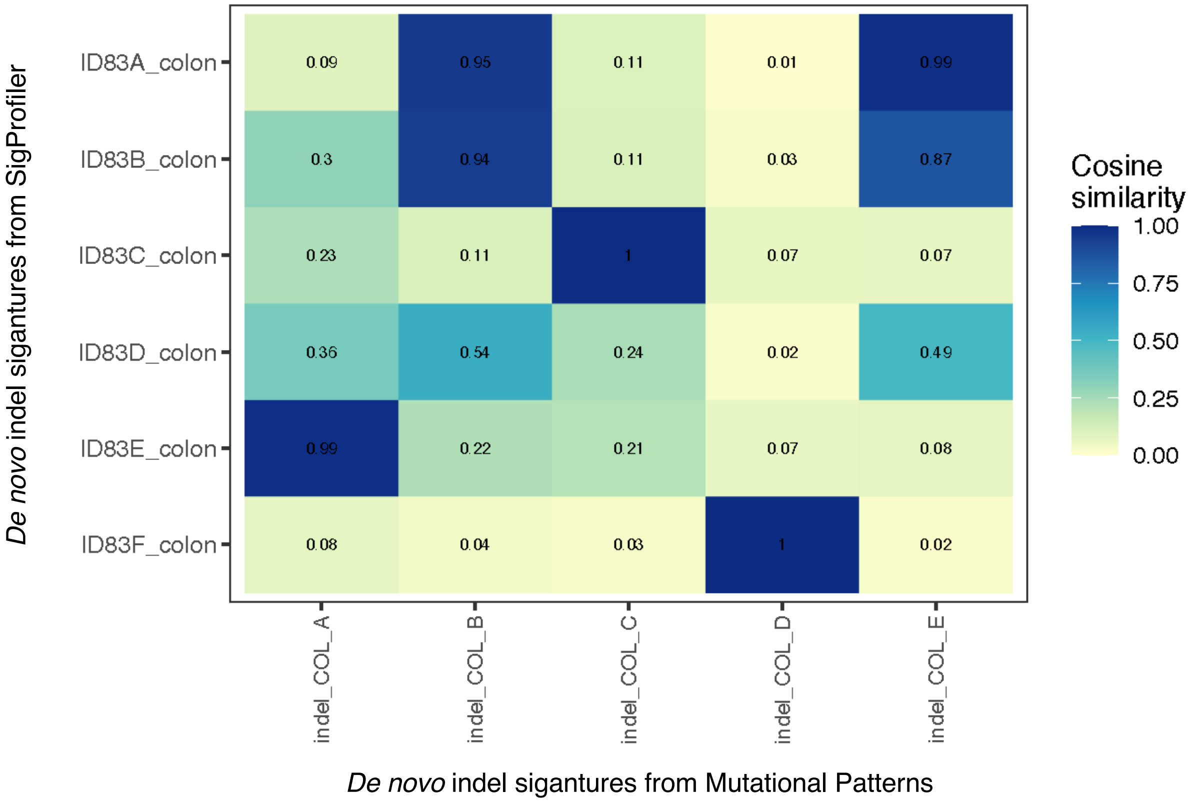

Supplementary Figure 5. ***De novo*** **indel signature validation in colorectal tissue.** (a) Heatmap showing the cosine similarity scores for each *de novo* extracted colorectal indel signature from Mutational Patterns with the COSMIC reference indel signatures. (b) Heatmap showing the cosine similarity scores for each *de novo* extracted SBS colorectal signature from Mutational Patterns with the *de novo* extracted SBS colorectal signature from SigProfiler.

Supplementary figure 6

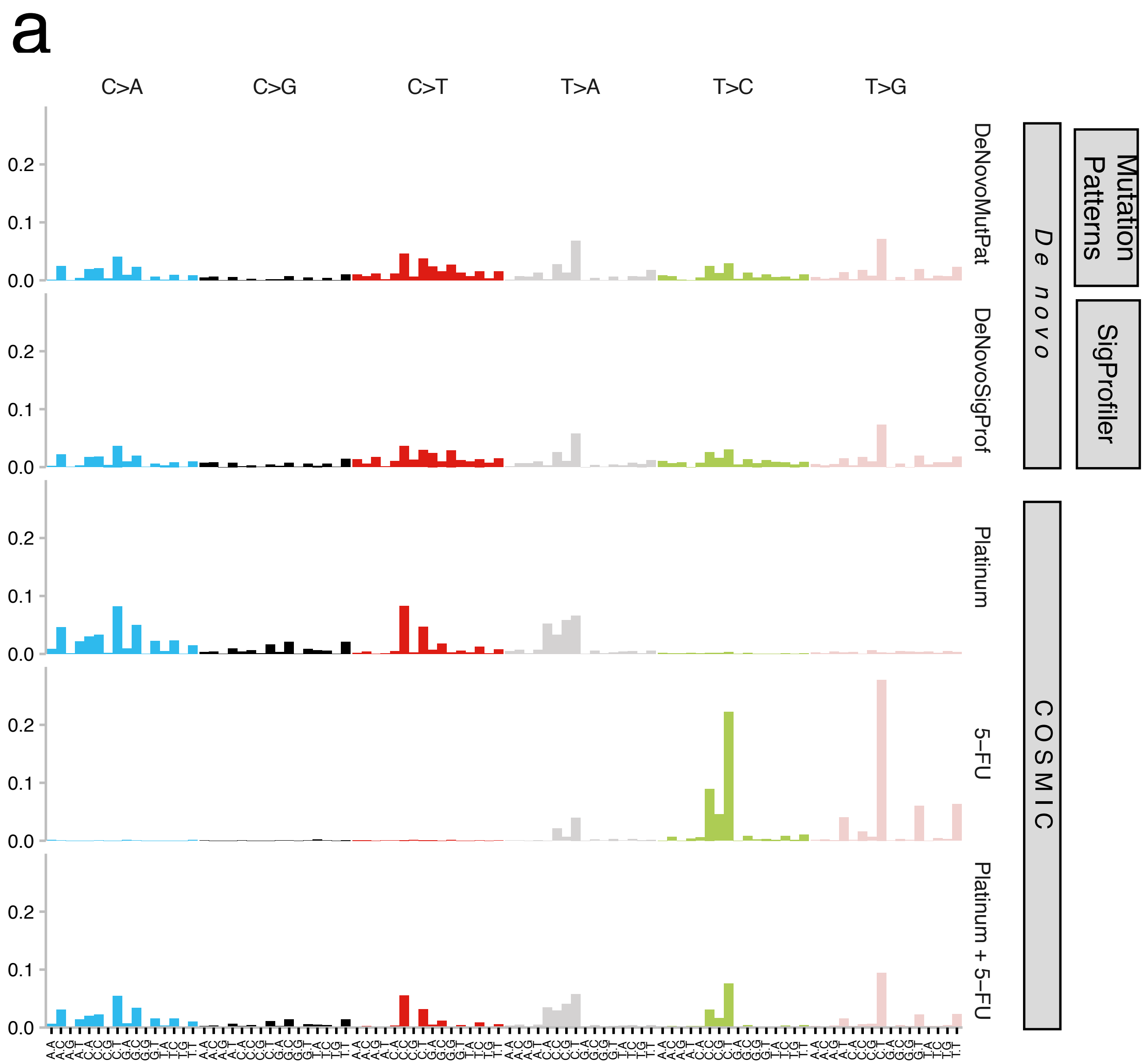

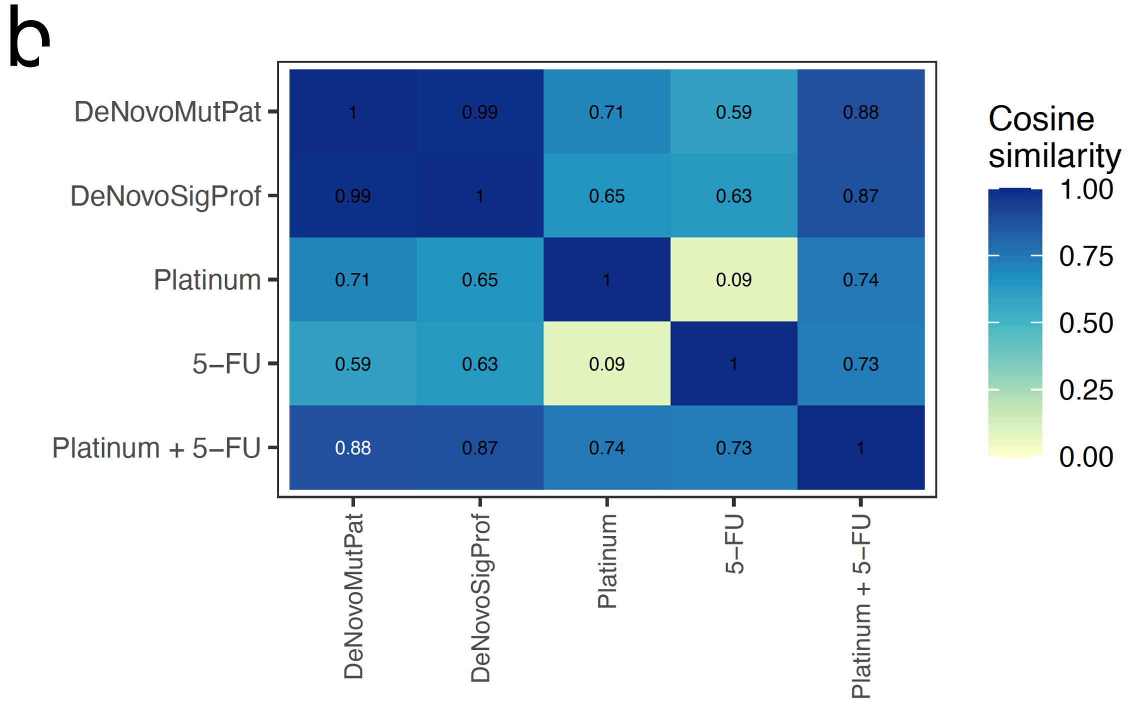

Supplementary Figure 6. Chemotherapy-induced SBS signature validation in colorectal tissue. (a) Mutational profiles (relative probability of each mutation context) of the COSMIC 5-FU signature (SBS17), COSMIC platinum signature (SBS35), the mixture 5-FU (SBS17) and platinum (SBS35) signature, the distinct *de novo* SBS signature from Mutational Patterns and the distinct *de novo* SBS signature from SigProfiler. (b) Heatmap showing the cosine similarity scores for the indicated signatures.

Supplementary figure 7

a

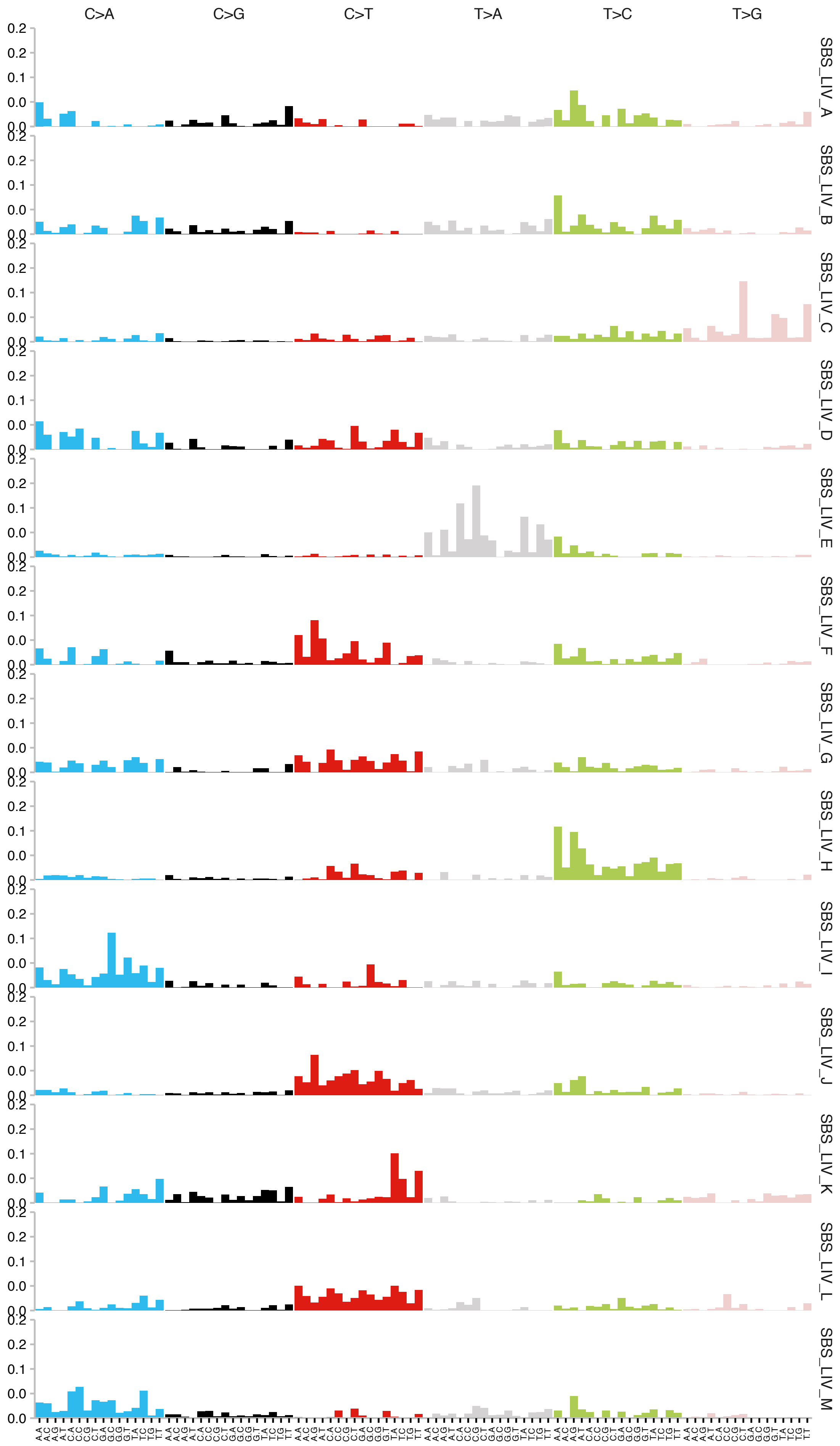

Supplementary Figure 7. *De novo* signatures in treated and untreated liver. (a) Mutational profiles (relative probability of each mutation context) of the thirteen SBS *de novo* signatures (no indel signatures could be extracted), extracted with Mutational Patterns, from treated and untreated liver tissue. The signatures were extracted on mutation data from 7 treated colorectal donors (established in this study),8 untreated donors[1], and a large public mutation dataset of healthy and diseases liver tissue from Brunner *et al*[3]*.* The number of *de novo* signatures for the indicated mutation type was chosen by comparing the *de novo* extracted signatures with COSMIC reference database using a cosine similarity score of 0.8 and by manual comparison with signatures from Brunner *et al*., (mutation profiles of the healthy liver signatures not available). Note that none of the *de novo* extracted SBS liver signatures shares any similarity with 5-FU (SBS17) or platinum (SBS35, SBS31) signatures except for SBS_LIV_C which shows a cosine similarity score of 0.79 with COSMIC17b and 0.74 with 5-FU signature.

Supplementary figure 8

a

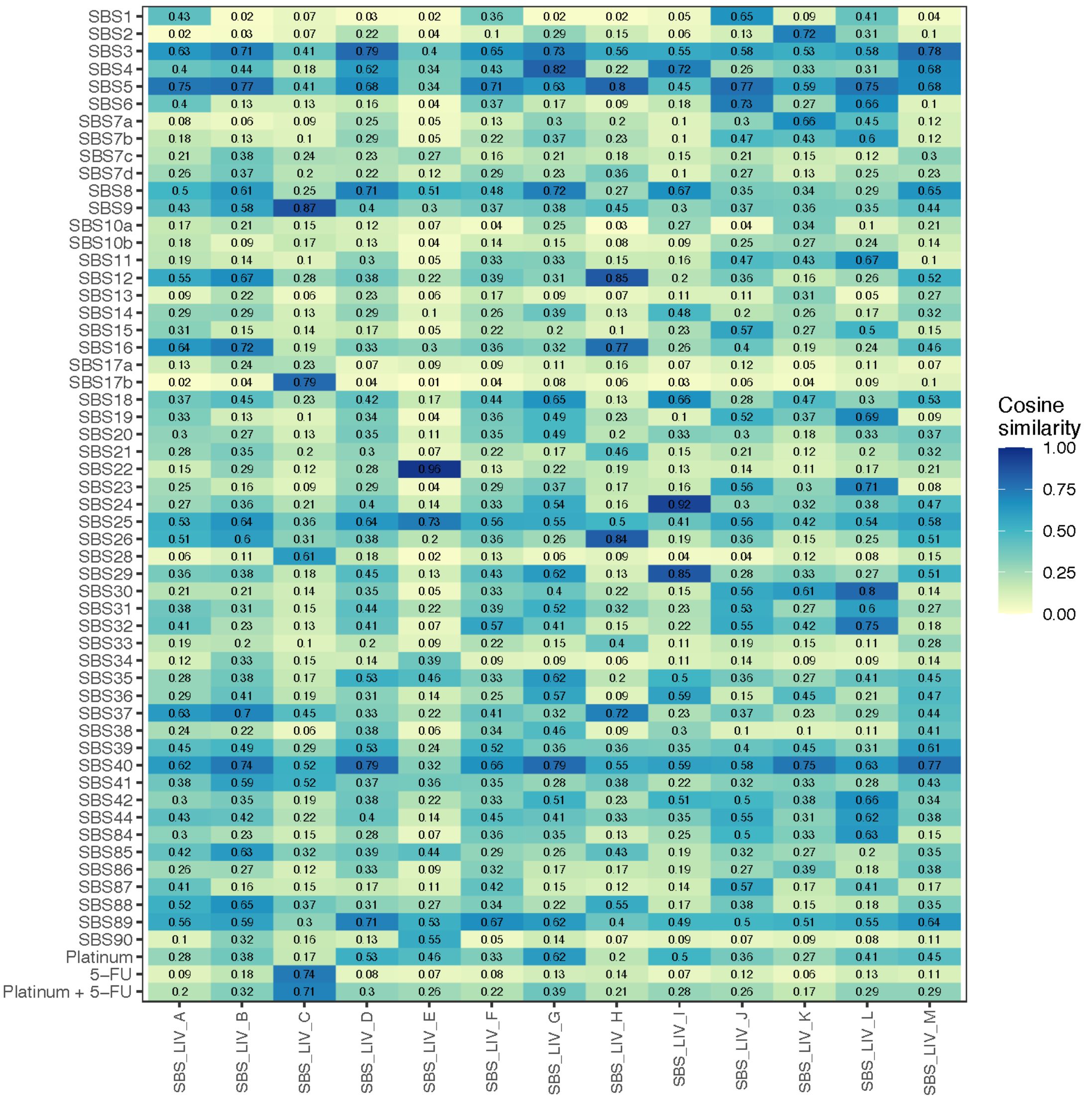

b

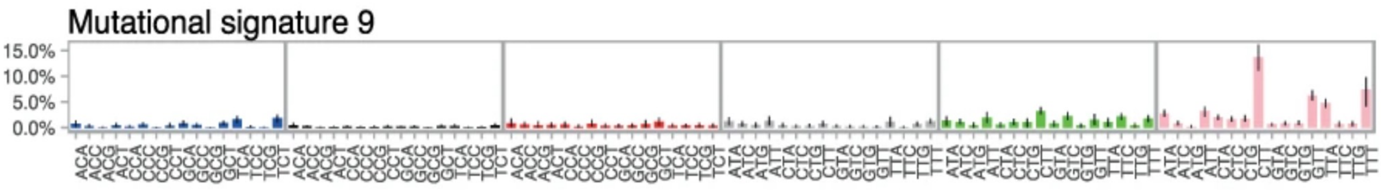

c

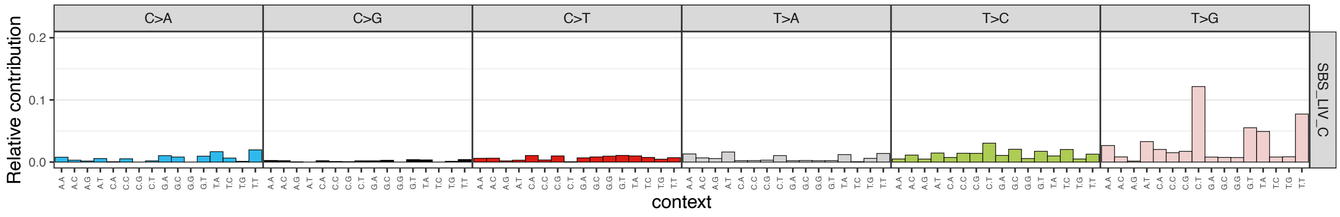

Supplementary Figure 8. ***De novo*** **SBS signature validation in liver tissue**

(a) Heatmap showing the cosine similarity scores for the *de novo* extracted liver SBS signature from Mutational Patterns with the COSMIC reference signatures. (b) SBS_LIV_C is also observed in one patient in the study of Brunner[3], which was linked to the process of somatic hypermutation in B lymphocytes. (c) Mutational profile of SBS_LIV_C for comparison.

Supplementary figure 9

a

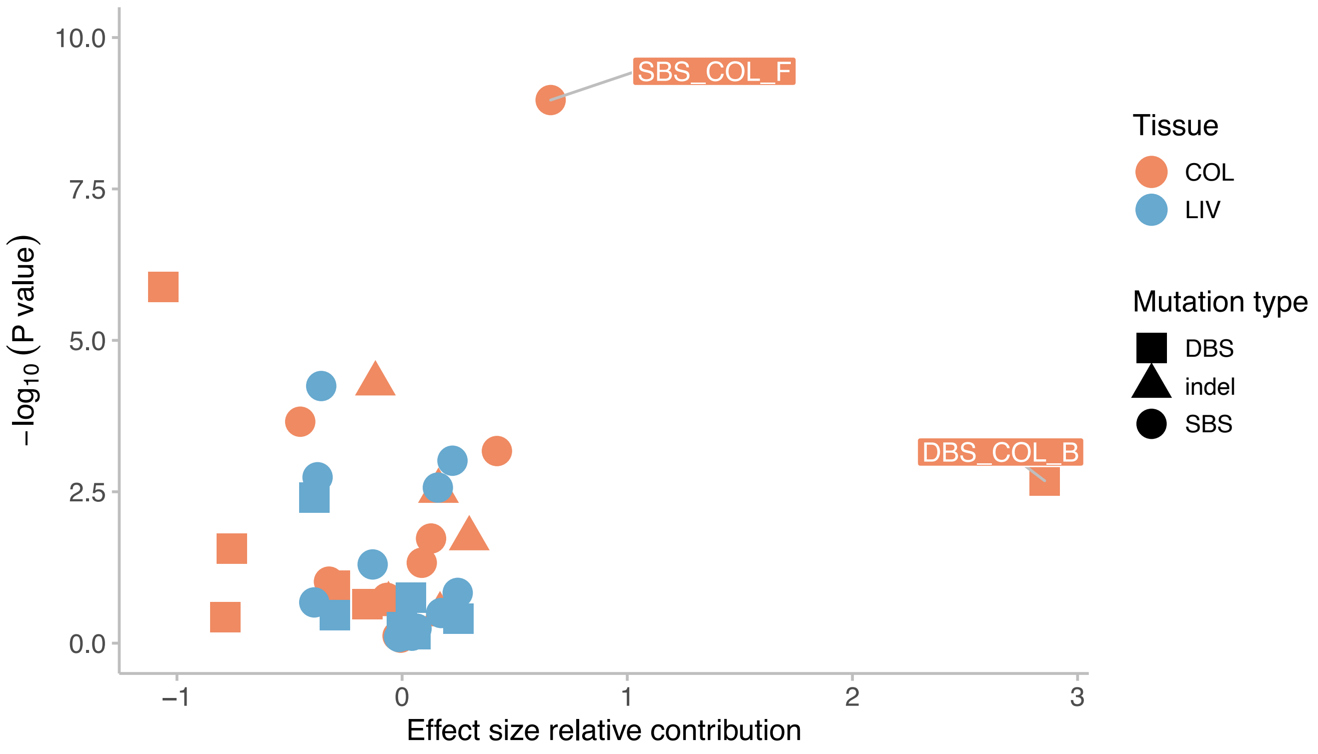

Supplementary Figure 9. Treatment-associated *de novo* mutational signatures in colorectal and liver. Each data point represents the significance (*y*-axis) and effect size (*x*-axis) of a Wilcoxon rank-sum test (two-sided) of the mutational signature contribution between treated and untreated ASCs. The color represents the tissue type and the indicated mutation type is given by the shape of each dot. SBS_COL_F shows a high significance whereas DBS_COL_B bears a strong effect size. Signatures not related to anti-cancer therapies cluster together including all liver signatures.

Supplementary figure 10

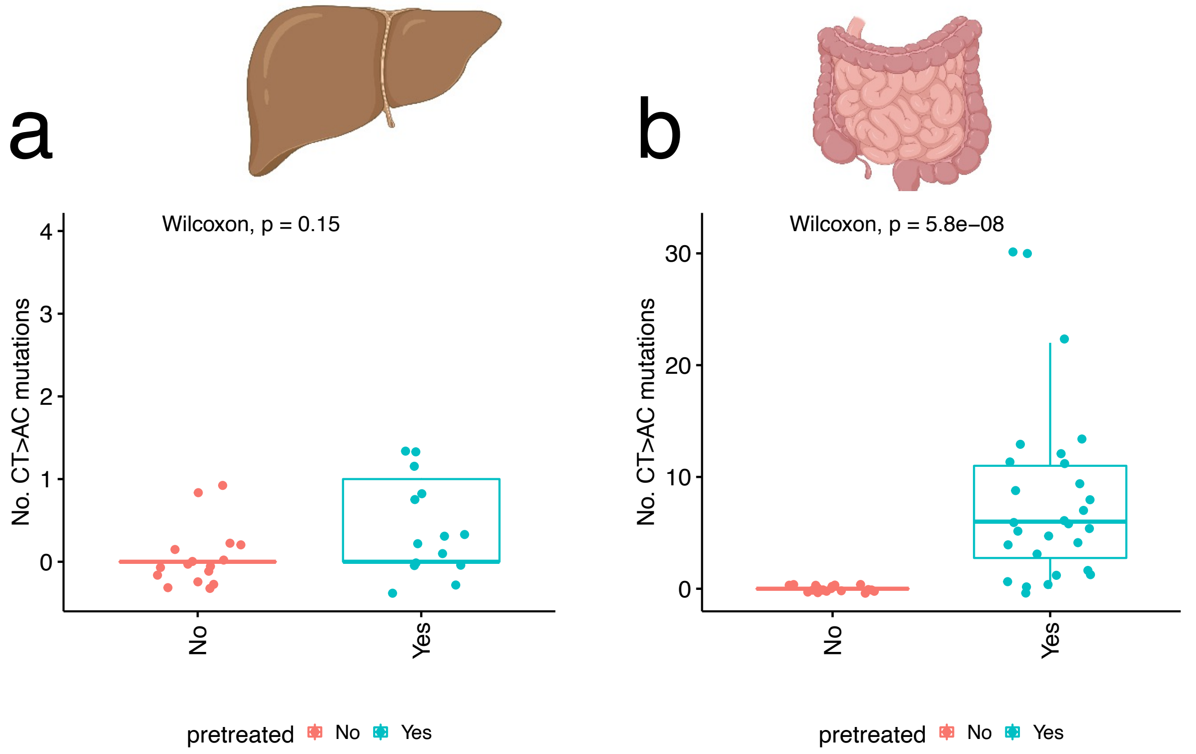

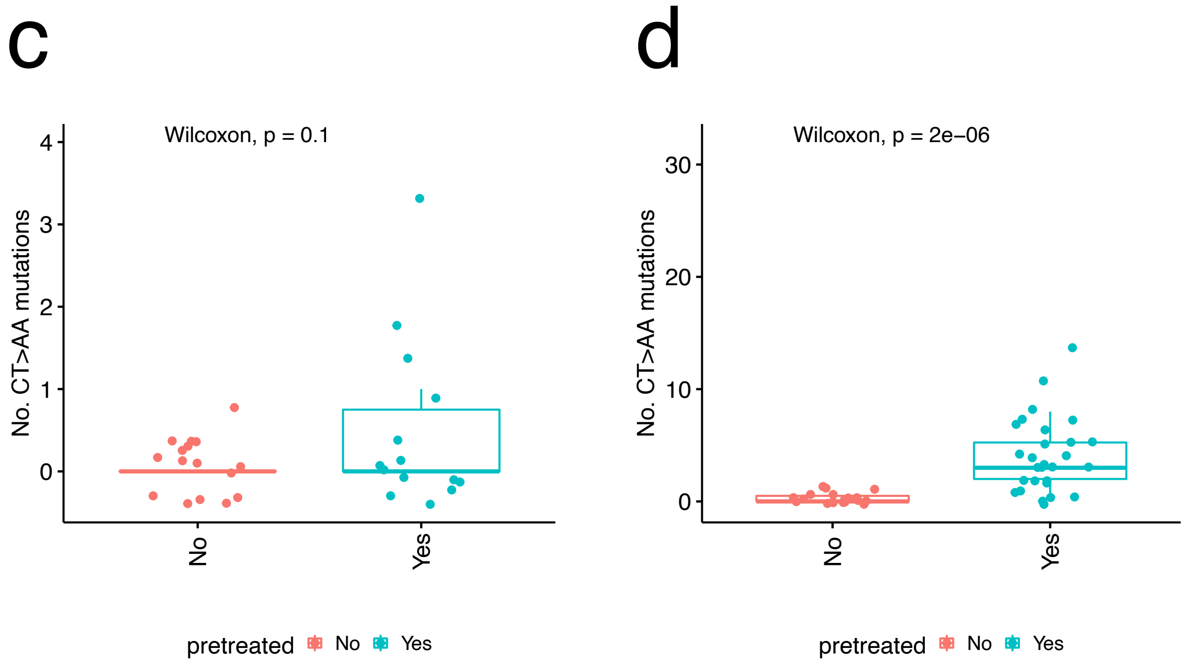

Supplementary Figure 10. Platinum-induced DBS mutations in colorectal and liver tissue. Box-and whisker plots showing the CT>AT (a, b) and CT>AA (c, d) DBS mutational count between treated (*n* = 14) and (*n* = 16) untreated liver (a ,c), and treated (*n* = 28) and (*n* = 19) untreated colorectal (b, d) ASCs. For all plots, a two-sided Wilcoxon rank-sum test between every cohort was performed and the P-value is illustrated at the top of the plots. The box in each boxplot delimits the first and third quartiles of the distribution (with a line representing the median); the whiskers delimit the lowest data point above the first quartile minus 1.5 times the interquartile distance and the highest data point below the third quartile plus 1.5 times the interquartile distance.

Supplementary figure 11

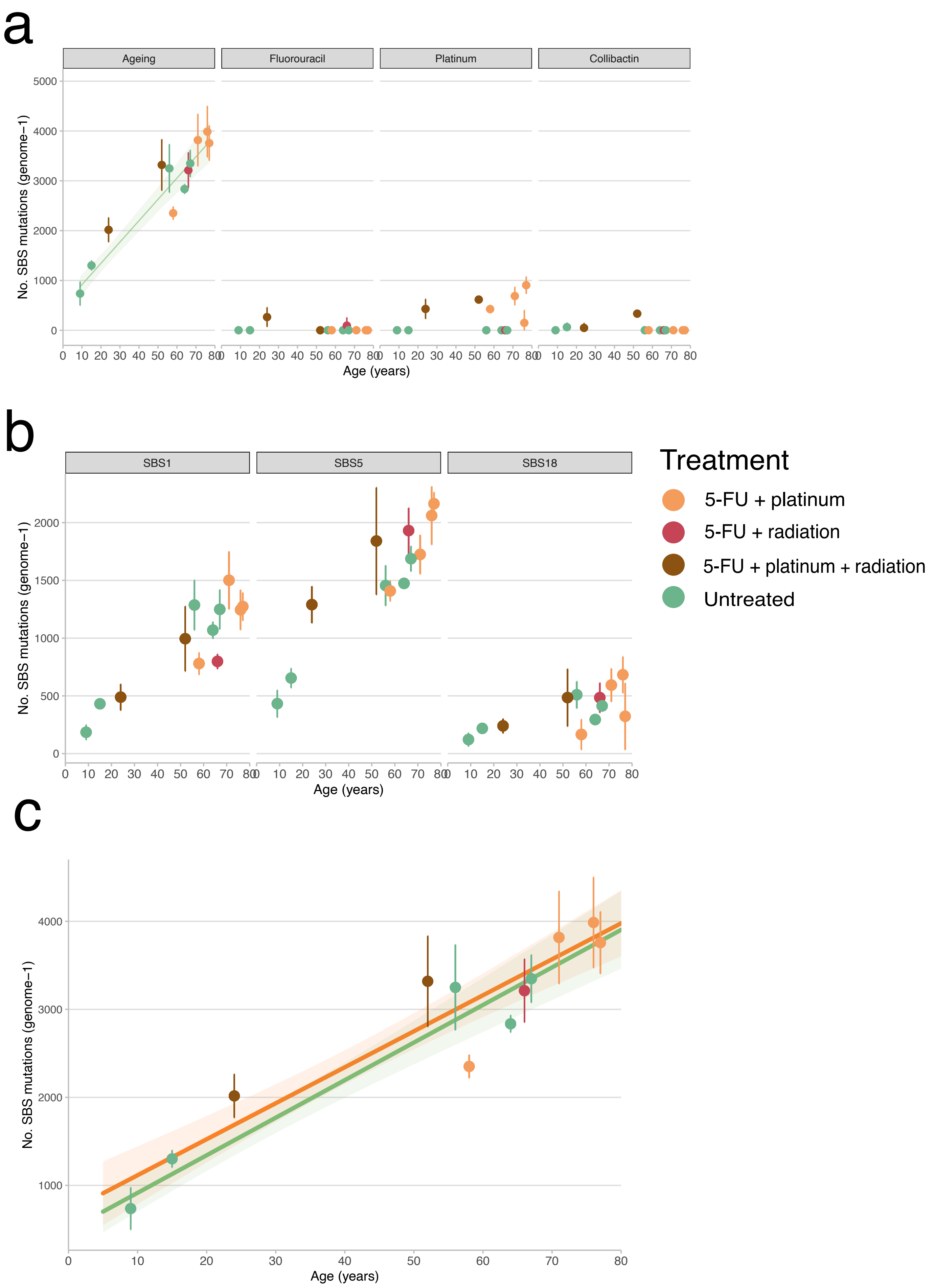

Supplementary Figure 11. Mutational signature contribution in colorectal ASCs. (a) Absolute contribution of environmentally induced mutational signatures plotted against the age of the donors from treated and untreated colorectal ASC. Colibactin-induced mutations[47] are also included (b) Absolute contribution of each age-related mutational signature plotted against the age of the donors from treated and untreated colorectal ASC. (a, b) Each data point represents the mean SBS load per donor and error bars represent standard deviation. The number of sequenced ASC per donor (*n*) varies from 1 up to 6 samples and is listed in Figure 1a. The color of each point depicts the treatment history. The cohort size for treated and untreated colorectal ASCs are respectively 6 and 7 donors. The green lines display the expected SBS load by age calculated from untreated ASCs using a bootstrapped linear mixed effects model approach. The shaded green area represents the standard deviation of the LMM by age. (c) Linear mixed model on untreated (green) and CapOx-treated (orange) colorectal ASCs showing no difference in age-related mutation accumulation. Untreated colorectal ASCs accumulate 43 mutations per year (95% CI: 27-60) and CapOx-treated colorectal ASCs 43 mutations per year (95% CI: 31-55). The 9- and 15-year-old donor were included in both LMM approaches to preserve age-related determinants at young age.

Supplementary figure 12

a

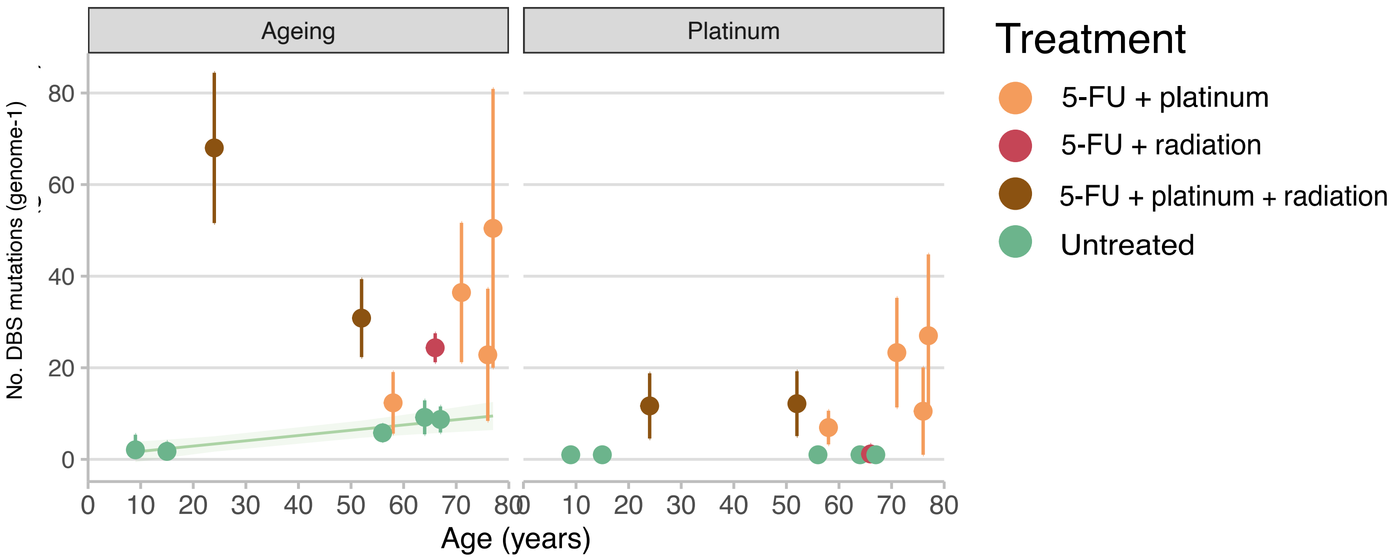

Supplementary Figure 12. DBS signature contribution in colorectal ASCs. Absolute contribution of the DBS mutational signatures plotted against the age of the donors from treated and untreated colorectal ASC. Each data point represents the mean DBS burden per donor and error bars represent standard deviation. The color of each point depicts the treatment history. The cohort size for treated and untreated colorectal ASCs are respectively 6 and 7 donors. The green lines display the expected DBS load calculated from untreated ASCs using a bootstrapped linear mixed effects model approach. The shaded green area represents the standard deviation of the LMM by age.

Supplementary figure 13

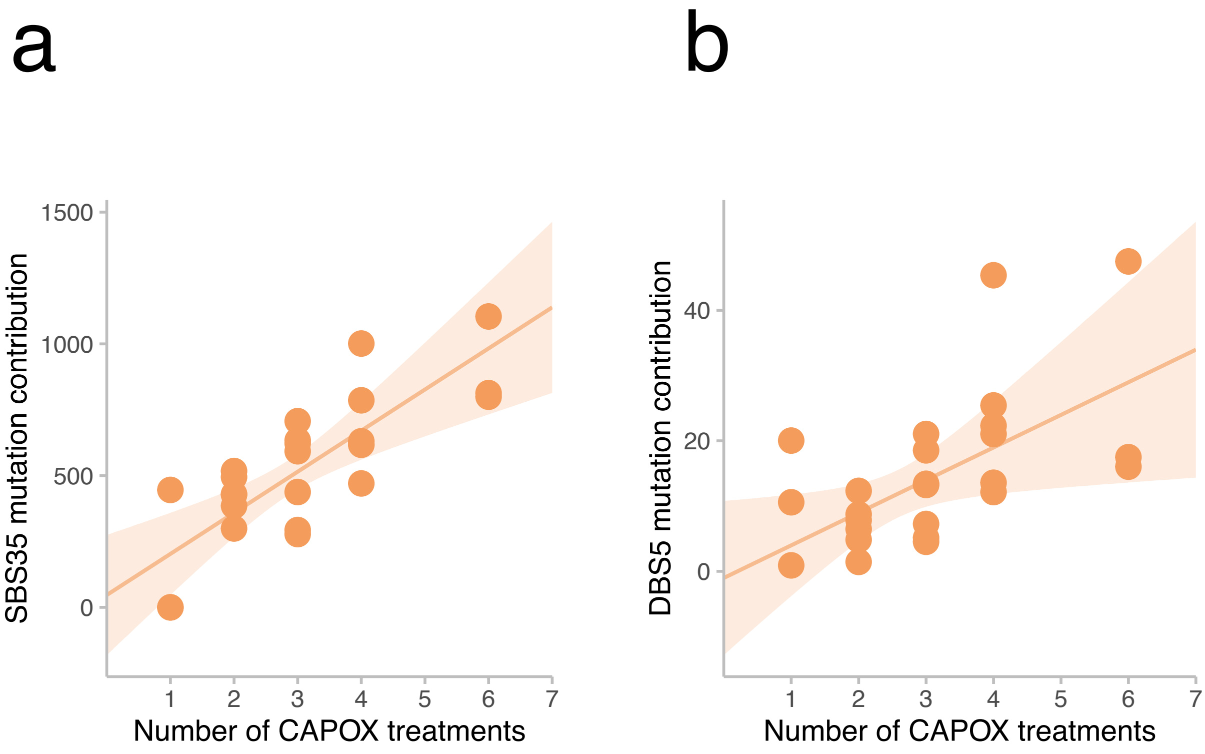

Supplementary Figure 13. CapOx treatments in relation to platinum mutation loads. Linear mixed model analysis on the number of CapOx treatments (*x* axis) to the (a) platinum SBS35 mutation contribution and (b) platinum DBS5 mutation contribution. Each CapOx cycle induces an additional 105 (±33 SE) SBS and 4 (±2 SE) DBS mutations (t-test linear mixed model; P_{SBS}=0.03; P_{DBS}=0.09). The shaded area represents the standard deviation of the LMM.

Supplementary figure 14

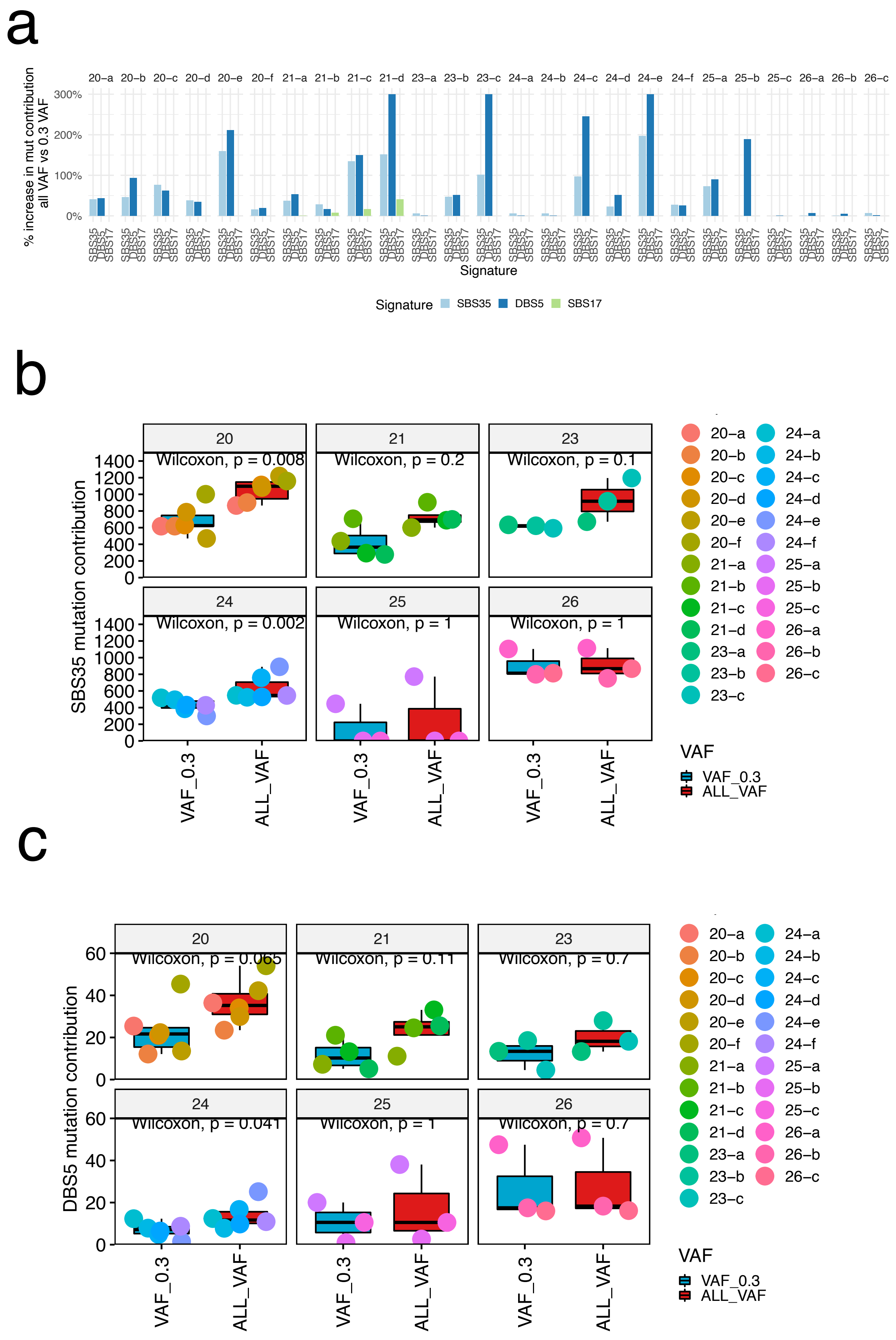

Supplementary Figure 14. Subclonal therapy-related mutations. (a) Barplot showing the increase in mutation contribution between 30% VAF filtered samples and samples without subclonal filtering. Sample IDs are presented at the top while the mutational signature is indicated by the color of each bar. (b,c) Box-and whisker plot indicating the absolute mutation contribution of COSMIC SBS35 (b) and DBS5 (c) between 30% VAF filtered samples and samples without subclonal filtering. The number of ASC per donor (*n*) for each barplot are shown in each figure as single dots. The box in each boxplot delimits the first and third quartiles of the distribution (with a line representing the median); the whiskers delimit the lowest data point above the first quartile minus 1.5 times the interquartile distance and the highest data point below the third quartile plus 1.5 times the interquartile distance.

Supplementary figure 15

a

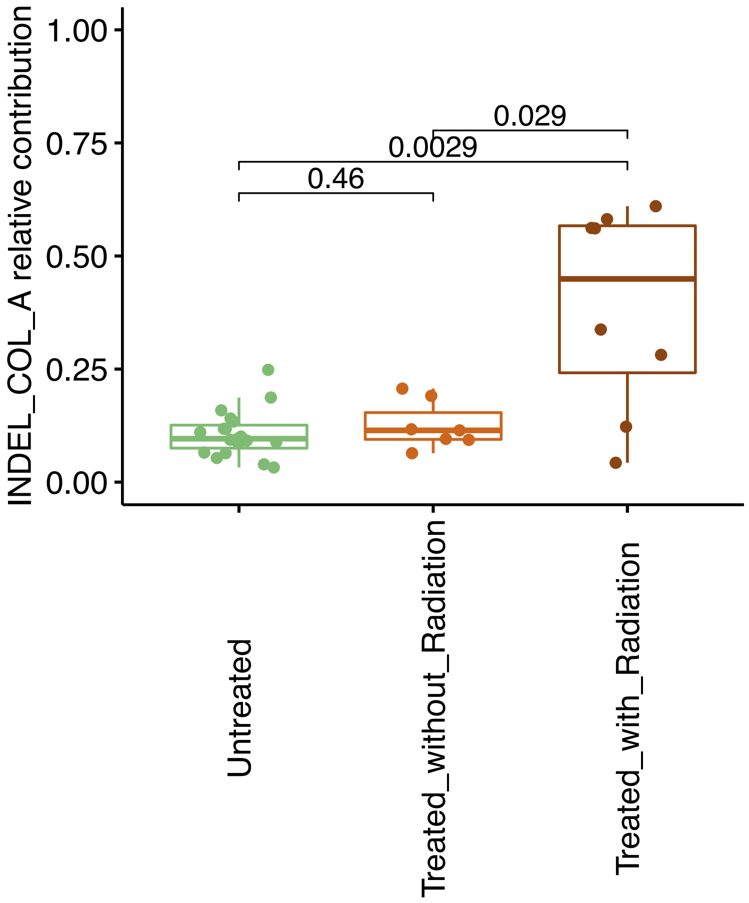

Supplementary Figure 15. Radiotherapy-induced indels in colorectal ASCs. Box-and whisker plot indicating the absolute mutation contribution of the *de novo* INDEL_COL_A indel signature between chemo-treated ASCs with (n = 10) and without (n = 18) radiotherapy and untreated (n = 19) colorectal ASCs. The box-and whiskers plots display the first and the third quartiles (top and bottom of the box), the median (vertical line inside the box), the extremes (whiskers) and the single data points (single dots). A two-sided Wilcoxon rank-sum test between every cohort was performed and the p-value is illustrated at the top of the plots.

Supplementary figure 16

a

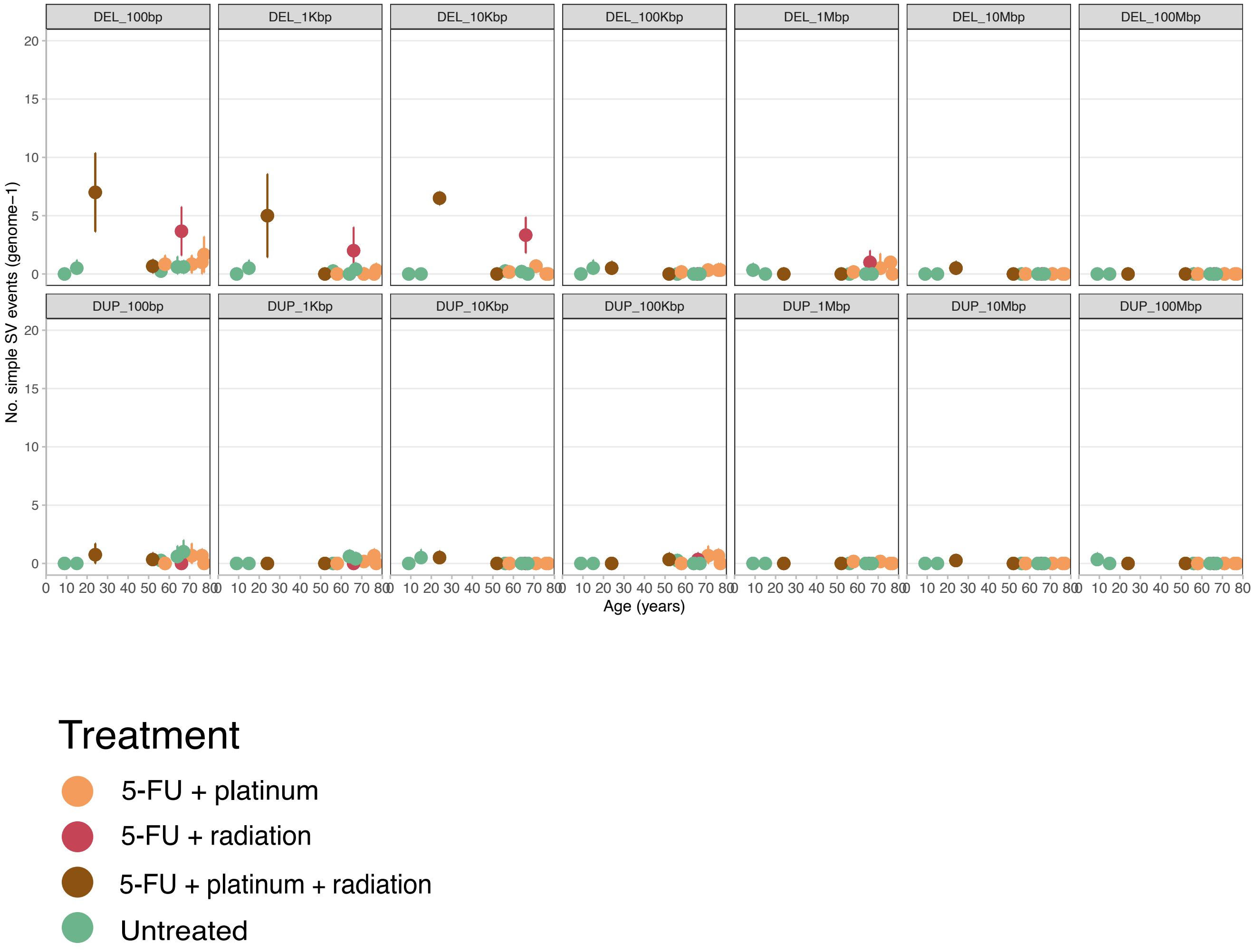

Supplementary Figure 16. Overview of structural mutation landscape in colorectal ASCs. Each box represents a structural variation type split by type and length. Each dot represents its mean mutation contribution (*y* axis) for each donor as a function of age (*x* axis). The error bars represent standard deviation and the color of each point depicts the treatment history. The number of ASC per donor (*n*) for each barplot are shown in each figure as single dots.

Supplementary figure 17

a

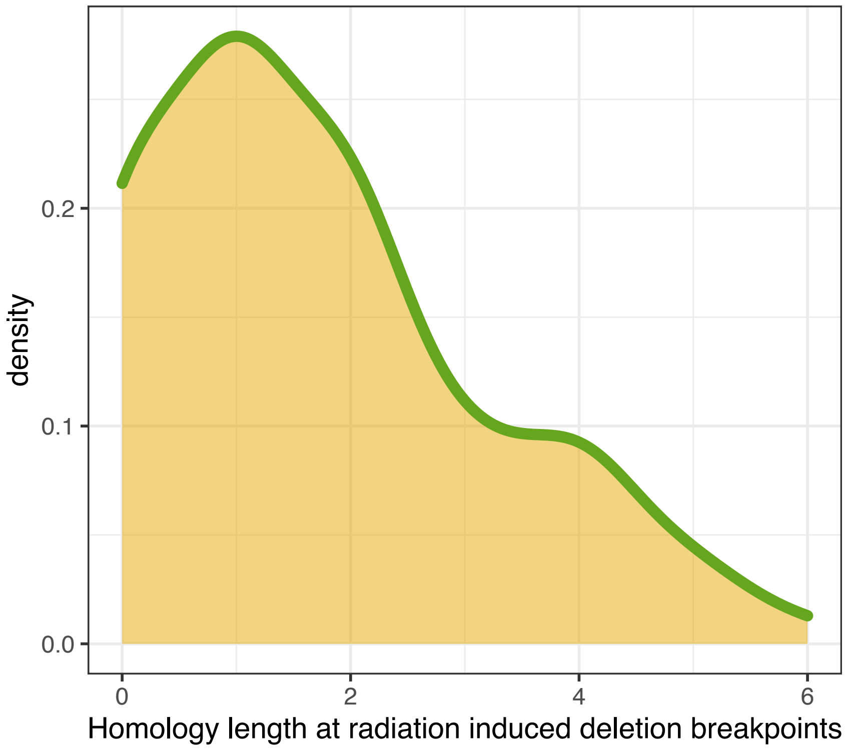

Supplementary Figure 17. Microhomology contexts of structural deletions. Microhomology contexts of the structural deletions obtained from 3 donors treated with radiotherapy.

Supplementary table 1

Donor_name	Donor_id	sample_id	Age	Sampling Tissue	Prim_tumor_sublocation	Origin_prim_tumor	Origin_metastatis_tumor	Healthy_tissue_sublocation	Treatment	CAPOX_treatments	Days_between_end_treatment_and_biopsy	Treatment_duration
PATIENT1	20	20-a	71	Colon	Descending colon	Colon	NA	Descending colon	5-FU+platinum	4	45	75
PATIENT1	20	20-b	71	Colon	Descending colon	Colon	NA	Descending colon	5-FU+platinum	4	45	75
PATIENT1	20	20-c	71	Colon	Descending colon	Colon	NA	Descending colon	5-FU+platinum	4	45	75
PATIENT1	20	20-d	71	Colon	Descending colon	Colon	NA	Descending colon	5-FU+platinum	4	45	75
PATIENT1	20	20-e	71	Colon	Descending colon	Colon	NA	Descending colon	5-FU+platinum	4	45	75
PATIENT1	20	20-f	71	Colon	Descending colon	Colon	NA	Descending colon	5-FU+platinum	4	45	75
PATIENT2	21	21-a	24	Colon	Rectum	Colon	NA	Rectum	5-FU+platinum+radiation	3	30	77
PATIENT2	21	21-b	24	Colon	Rectum	Colon	NA	Rectum	5-FU+platinum+radiation	3	30	77
PATIENT2	21	21-c	24	Colon	Rectum	Colon	NA	Rectum	5-FU+platinum+radiation	3	30	77
PATIENT2	21	21-d	24	Colon	Rectum	Colon	NA	Rectum	5-FU+platinum+radiation	3	30	77
PATIENT3	22	22-a	66	Colon	Rectum	Colon	NA	Rectum	5-FU+radiation	NA	NA	34
PATIENT3	22	22-b	66	Colon	Rectum	Colon	NA	Rectum	5-FU+radiation	NA	NA	34
PATIENT3	22	22-c	66	Colon	Rectum	Colon	NA	Rectum	5-FU+radiation	NA	NA	34
PATIENT4	23	23-a	52	Colon	Rectum	Colon	NA	Rectum	5-FU+platinum+radiation	3	90	51
PATIENT4	23	23-b	52	Colon	Rectum	Colon	NA	Rectum	5-FU+platinum+radiation	3	90	51
PATIENT4	23	23-c	52	Colon	Rectum	Colon	NA	Rectum	5-FU+platinum+radiation	3	90	51
PATIENT5	24	24-a	58	Colon	Sigmoid	Colon	NA	Sigmoid	5-FU+platinum	2	30	41
PATIENT5	24	24-b	58	Colon	Sigmoid	Colon	NA	Sigmoid	5-FU+platinum	2	30	41
PATIENT5	24	24-c	58	Colon	Sigmoid	Colon	NA	Sigmoid	5-FU+platinum	2	30	41
PATIENT5	24	24-d	58	Colon	Sigmoid	Colon	NA	Sigmoid	5-FU+platinum	2	30	41
PATIENT5	24	24-e	58	Colon	Sigmoid	Colon	NA	Sigmoid	5-FU+platinum	2	30	41
PATIENT5	24	24-f	58	Colon	Sigmoid	Colon	NA	Sigmoid	5-FU+platinum	2	30	41
PATIENT6	50	50-a	68	Liver	Colon	Colon	Liver	Colon	5-FU+platinum	6	1095	NA
PATIENT6	50	50-b	68	Liver	Colon	Colon	Liver	Colon	5-FU+platinum	6	1095	NA
PATIENT7	51	51-a	72	Liver	Colon	Colon	Liver	Colon	5-FU+platinum	7	730	147
PATIENT7	51	51-b	72	Liver	Colon	Colon	Liver	Colon	5-FU+platinum	7	730	147
PATIENT8	52	52-a	45	Liver	Colon	Colon	Liver	Colon	5-FU+radiation	NA	30	34
PATIENT8	52	52-b	45	Liver	Colon	Colon	Liver	Colon	5-FU+radiation	NA	30	34
PATIENT9	53	53-b	61	Liver	Colon	Colon	Liver	Colon	5-FU+platinum	2	210	21
PATIENT10	54	54-a	60	Liver	Colon	Colon	Liver	Colon	5-FU+platinum	4	30	NA
PATIENT10	54	54-b	60	Liver	Colon	Colon	Liver	Colon	5-FU+platinum	4	30	NA
PATIENT10	54	54-c	60	Liver	Colon	Colon	Liver	Colon	5-FU+platinum	4	30	NA
PATIENT11	55	55-a	48	Liver	Colon	Colon	Liver	Colon	5-FU+platinum	12	630	77
PATIENT12	25	25-a	76	Colon	Descending colon	Colon	NA	Descending colon	5-FU+platinum	1	45	14
PATIENT12	25	25-b	76	Colon	Descending colon	Colon	NA	Descending colon	5-FU+platinum	1	45	14
PATIENT12	25	25-c	76	Colon	Descending colon	Colon	NA	Descending colon	5-FU+platinum	1	45	14
PATIENT13	26	26-a	77	Colon	Sigmoid	Colon	NA	Sigmoid	5-FU+platinum	6	15	NA
PATIENT13	26	26-b	77	Colon	Sigmoid	Colon	NA	Sigmoid	5-FU+platinum	6	15	NA
PATIENT13	26	26-c	77	Colon	Sigmoid	Colon	NA	Sigmoid	5-FU+platinum	6	15	NA
PATIENT14	56	56-a	70	Liver	Colon	Colon	Liver	Colon	5-FU+platinum	4	75	NA
PATIENT14	56	56-b	70	Liver	Colon	Colon	Liver	Colon	5-FU+platinum	4	75	NA
PATIENT14	56	56-c	70	Liver	Colon	Colon	Liver	Colon	5-FU+platinum	4	75	NA

Supplementary Note 1: Power Analysis

In this study we relied on a sample size set similar to other studies[1,4,5,6,7] that also used healthy tissue-derived organoids to investigate mutation accumulation. To further explore the sample size of the current study, we have conducted a power analysis as in our previous study[7]. For this, we first measured the Cohen's D to compute the standardised mean difference of the mutation load between platinum treated and untreated donors. Using the cohen.D function from effsize R package, we found a normalized effect size of 1.51. A similar normalized effect size (Cohen's D = 1.52) was observed when we assessed the excess in mutation load (observed mut load - predicted mut load using linear mixed model regression analysis) between platinum treated and untreated donors.

To assess the reliability of measured Cohen's D scores on mutation load given our number of samples, we performed a power analysis using the pwr.t2n.test() function of the pwr R package to calculate hypothetical detectable effect sizes using a significance level of 0.05 and a power of 0.8. It is generally accepted that power should be approximately 80%. Given that we have 5 untreated patients and 6 treated patients, we expect to be able to detect an effect size (Cohen's D) of 1.6 which is approximately equal to the computed Cohen's D values on the mutation load and excess mutation load. Thus, the sample sizes in our study would allow us to detect effect sizes as hypothetical detectable effect sizes with a power score of 80%.

Similarly, we applied this approach to the platinum and 5-FU mutation contributions. Using the SBS35 and SBS17 mutation contributions, we obtained Cohen's D scores of 2.7 (large) and 0.7 (medium) for platinum and 5-FU mutation contributions, respectively, between the treated and untreated donor group. The Cohen's D score on the platinum DBS (DBS5) contribution was 2.5, which is similar to the SBS35 mutation contribution. Thus, the normalised effect size for platinum mutation load is well beyond the hypothetical detectable effect size given the number of samples (1.6), but not for 5-FU mutation contribution. This is in line with our observations that 5-FU mutations were only detected in few samples of 2 (of 7) donors.

Lastly, we found that the Cohen's D score for radiation-induced indels (ID8) between untreated and radiation-treated donors was also higher (ie 1.8) than the hypothetical detectable effect size with higher power.

References

1. Blokzijl, F. et al. Tissue-specific mutation accumulation in human adult stem cells during life. Nature 538, 260–264 (2016).
2. Lee-Six, H. et al. The landscape of somatic mutation in normal colorectal epithelial cells. Nature 574, 532–537 (2019).
3. Brunner, S. F. et al. Somatic mutations and clonal dynamics in healthy and cirrhotic human liver. Nature 574, 538–542 (2019).
4. Jager, M. et al. Deficiency of nucleotide excision repair is associated with mutational signature observed in cancer. Genome Res. 29, 1067–1077 (2019).
5. Drost, J. et al. Use of CRISPR-modified human stem cell organoids to study the origin of mutational signatures in cancer. Science 358, 234–238 (2017).
6. Kuijk, E. et al. The mutational impact of culturing human pluripotent and adult stem cells. Nat. Commun. 11, 2493 (2020).
7. Nguyen, L. et al. Precancerous liver diseases do not cause increased mutagenesis in liver stem cells. Commun Biol 4, 1301 (2021).
