# Supplementary material for: Common anti-cancer therapies induce somatic mutations in stem cells of healthy tissue
Source: Nat Commun. 2022 Oct 7;13:5915. doi: 10.1038/s41467-022-33663-5 (PMC9546852; doi:10.1038/s41467-022-33663-5)
Supplement: Supplementary file 3 — Reporting Summary [file 41467_2022_33663_MOESM3_ESM.pdf]

nature portfolio

Corresponding author(s): Edwin Cuppen, Arne Van Hoeck

Last updated by author(s): 11-04-2022

Reporting Summary

Nature Portfolio wishes to improve the reproducibility of the work that we publish. This form provides structure for consistency and transparency in reporting. For further information on Nature Portfolio policies, see our Editorial Policies and the Editorial Policy Checklist.

Statistics

For all statistical analyses, confirm that the following items are present in the figure legend, table legend, main text, or Methods section.

n/a	Confirmed	
☐	☒	The exact sample size (*n*) for each experimental group/condition, given as a discrete number and unit of measurement
☐	☒	A statement on whether measurements were taken from distinct samples or whether the same sample was measured repeatedly
☐	☒	The statistical test(s) used AND whether they are one- or two-sided<br>*Only common tests should be described solely by name; describe more complex techniques in the Methods section.*
☒	☐	A description of all covariates tested
☐	☒	A description of any assumptions or corrections, such as tests of normality and adjustment for multiple comparisons
☐	☒	A full description of the statistical parameters including central tendency (e.g. means) or other basic estimates (e.g. regression coefficient) AND variation (e.g. standard deviation) or associated estimates of uncertainty (e.g. confidence intervals)
☐	☒	For null hypothesis testing, the test statistic (e.g. *F*, *t*, *r*) with confidence intervals, effect sizes, degrees of freedom and *P* value noted<br>*Give P values as exact values whenever suitable.*
☒	☐	For Bayesian analysis, information on the choice of priors and Markov chain Monte Carlo settings
☒	☐	For hierarchical and complex designs, identification of the appropriate level for tests and full reporting of outcomes
☐	☒	Estimates of effect sizes (e.g. Cohen's *d*, Pearson's *r*), indicating how they were calculated

Our web collection on statistics for biologists contains articles on many of the points above.

Software and code

Policy information about availability of computer code

Data collection	No software was used for data collection.
Data analysis	All code used for data analysis and code used is publicly available and can be found at https://github.com/UMCUGenetics/treatment_muts_healthy_tissue.<br>We used software from the following external resources: https://github.com/hartwigmedical/pipeline, https://github.com/hartwigmedical/hmftools, https://github.com/UMCUGenetics/MutationalPatterns/, https://github.com/im3sanger/dndscv, https://github.com/AlexandrovLab/SigProfilerExtractor

For manuscripts utilizing custom algorithms or software that are central to the research but not yet described in published literature, software must be made available to editors and reviewers. We strongly encourage code deposition in a community repository (e.g. GitHub). See the Nature Portfolio guidelines for submitting code & software for further information.

Data

Policy information about availability of data

All manuscripts must include a data availability statement. This statement should provide the following information, where applicable:

- Accession codes, unique identifiers, or web links for publicly available datasets
- A description of any restrictions on data availability
- For clinical datasets or third party data, please ensure that the statement adheres to our policy

The human sequencing data of treated donor generated in this study have been deposited at the European Genome-phenome Archive (http://www.ebi.ac.uk/ega/) under accession numbers EGAS00001006042 [https://ega-archive.org/studies/EGAS00001006042]. Access can be obtained by UMCU DAC. Raw sequencing data of colorectal and liver ASCs from untreated donors were downloaded from EGA download portal under accession numbers EGAS00001001682 and

EGAS00001000881. We also included data from https://doi.org/10.1038/s41586-019-1672-7 and https://doi.org/10.1038/s41586-019-1672-7.The processed mutations in this study are provided in the Supplementary Information/Source Data file. The mutation calls from the sequencing data used in this study are available in the Zenodo database under accession code [https://doi.org/10.5281/zenodo.7057493].

Field-specific reporting

Please select the one below that is the best fit for your research. If you are not sure, read the appropriate sections before making your selection.

☒ Life sciences ☐ Behavioural & social sciences ☐ Ecological, evolutionary & environmental sciences

For a reference copy of the document with all sections, see nature.com/documents/nr-reporting-summary-flat.pdf

Life sciences study design

All studies must disclose on these points even when the disclosure is negative.

Sample size	Sample size were determined based on previous sequencing results (Blokwijl et al., Nature 2016)
Data exclusions	No data has been excluded in this study
Replication	Experiments were performed on biological replicates (mean of 3.2 ASC per donor) to verify the reproducibility of the experimental findings. In total 42 organoid lines were sequenced from 14 different donors that have been treated with chemo and/or radiation therapy. 35 organoid lines from 13 untreated donors were used as control
Randomization	From bulk cultures, random individual stem cells were used for clonal expansion.
Blinding	Not applicable to our study design, which is an unbiased approach where all the data is analyzed systematically using the same filtering pipelines independent of subjective interpretation.

Reporting for specific materials, systems and methods

We require information from authors about some types of materials, experimental systems and methods used in many studies. Here, indicate whether each material, system or method listed is relevant to your study. If you are not sure if a list item applies to your research, read the appropriate section before selecting a response.

Materials & experimental systems

n/a	Involved in the study	
☒	☐	Antibodies
☐	☒	Eukaryotic cell lines
☒	☐	Palaeontology and archaeology
☒	☐	Animals and other organisms
☐	☒	Human research participants
☒	☐	Clinical data
☒	☐	Dual use research of concern

Methods

n/a	Involved in the study	
☒	☐	ChIP-seq
☒	☐	Flow cytometry
☒	☐	MRI-based neuroimaging

Eukaryotic cell lines

Policy information about cell lines

Cell line source(s)	The healthy human small intestinal organoid line was obtained from UMC Utrecht with the approval for using of this material for research purposes following Biobanking protocol HUB-Cancer protocol.
Authentication	The organoids were whole genome sequenced to confirm identity.
Mycoplasma contamination	The organoid lines were negatively tested for mycoplasma contamination.
Commonly misidentified lines (See ICLAC register)	NA

Human research participants

Policy information about studies involving human research participants

Population characteristics	Liver and colon biopsy samples were obtained from colon cancer patients who have been treated with 5-FU and/or Oxaliplatin. The anonymous research participants were randomly included.

Recruitment	Recruitment was performed in collaboration with UPORT who organises the logistics of acquiring human and animal tissues for research.
Ethics oversight	Tissue samples from colorectal cancer patients were obtained from UMCU pathology department within the Biobanking protocol HUB-Cancer TCBIO, which was approved by the medical ethical committee of the University Medical Center Utrecht (UMCU). Written informed consent from the donors was obtained prior to acquisition of the specimen for research use in the present study.

Note that full information on the approval of the study protocol must also be provided in the manuscript.
